# In silico analysis of MALAT1 and H19 lncRNA interactions with the diabetes-related proteins ASK1 and YAP1

**DOI:** 10.1007/s40203-026-00734-0

**Published:** 2026-09-14

**Authors:** Leonardo Elías Cabrera-Nájera, Olga Nelly Rodríguez-Peña, Cesar Mateo Flores-Ortíz, Martín Palomar-Morales

**Affiliations:** 1 Laboratorio de Metabolismo de La Diabetes Mellitus, Unidad de Morfología y Función, Facultad de Estudios Superiores Iztacala, Universidad Nacional Autónoma de México, Tlalnepantla, Estado de México México; 2 Laboratorio de Biogeoquímica, Unidad de Biología, Tecnología y Prototipos (UBIPRO), Facultad de Estudios Superiores Iztacala, Universidad Nacional Autónoma de México, Tlalnepantla, Estado de México México; 3 Laboratorio Nacional en Salud, Facultad de Estudios Superiores Iztacala, Universidad Nacional Autónoma de México, Tlalnepantla, Estado de México México

**Keywords:** Allosteric regulation, Antagonists and inhibitors, Binding sites, Protein interaction domain, Protein interaction mapping, Diabetes mellitus

## Abstract

Dysregulation of RNA–protein signalling networks contributes to the progression of diabetes-associated complications, yet the structural mechanisms underlying lncRNA interactions with key signalling proteins remain largely unknown. This study investigates potential interaction interfaces between MALAT1–ASK1 and H19–YAP1 using a hypothesis-generating in silico approach. A computational pipeline integrating structure prediction, molecular docking, and molecular dynamics simulations was employed. Structural assessment showed high local confidence for both proteins and lncRNAs (PAE < 1 Å) but low global lncRNA folding reliability (pTM < 0.5), consistent with intrinsic structural flexibility. ASK1 and YAP1 exhibited multiple surface cavities and disordered regions involved in signalling regulation. Docking and simulations suggested MALAT1 binding to exposed ASK1 surfaces, while H19 was predicted to associate with YAP1 regions encompassing the TEAD-binding domain and conserved regulatory phosphorylation sites, including residues corresponding to Ser109, Ser127, and Thr119. Molecular dynamics indicated transient stabilisation with persistent conformational heterogeneity. These results identify structurally plausible RNA–protein interaction interfaces that overlap with known regulatory hotspots in YAP1. Overall, this work provides a framework for experimental validation of lncRNA-mediated regulation in diabetes-related signalling pathways.

## Introduction

Diabetes mellitus comprises a group of metabolic disorders, characterised by impaired carbohydrate metabolism, in which glucose utilisation is compromised, leading to increased gluconeogenesis and glycogenolysis and, consequently, chronic hyperglycaemia (ADA 2026). Diabetes is currently recognised as one of the leading causes of morbidity and mortality worldwide (GBD 2021 Diabetes Collaborators 2023), affecting approximately 589 million adults globally (Genitsaridi et al. 2025).

Uncontrolled diabetes mellitus is strongly associated with the development of microvascular and macrovascular complications (Zhao et al. 2026). In recent years, increasing evidence has demonstrated that disease progression is also influenced by molecular alterations involving regulatory non-coding RNAs, particularly microRNAs (miRNAs) and long non-coding RNAs (lncRNAs) (Yang et al. 2024), which participate in several stages of diabetic pathology.

lncRNAs have attracted considerable attention due to their pleiotropic and tissue-specific regulatory functions (Pasut et al. 2016; Zeng et al. 2023). These molecules participate in transcriptional, post-transcriptional and epigenetic regulation and can directly interact with proteins to modulate signalling pathways and cellular organisation (Arunkumar 2023; Bridges et al. 2021; Chen and Shan 2020; Sebastian-de la Cruz et al. 2021). Through these interactions, lncRNAs contribute to protein localisation and multiprotein complex assembly (Fernandes and Buchan 2021; Statello et al. 2021; Wang et al. 2025). However, despite increasing evidence supporting the involvement of lncRNAs in diabetes-related pathways, the structural interaction patterns between specific lncRNAs and diabetes-associated proteins remain poorly characterised.

Of the diabetes-associated lncRNAs, MALAT1 and H19 are among the most extensively studied due to their involvement in oxidative stress, inflammation, proliferation and metabolic dysregulation (Tanwar et al. 2021). In diabetes and its associated complications, MALAT1 (Metastasis-Associated Lung Adenocarcinoma Transcript 1) dysregulation has been associated with apoptosis (Abdulle et al. 2019), fibrosis and angiogenesis (Biswas et al. 2018; Geng et al. 2024), as well as pathological epithelial-to-mesenchymal transition (EMT) (Zhang et al. 2019). Furthermore, MALAT1 has been implicated in the regulation of oxidative stress through p38 MAPK signalling (Yang et al. 2018). As oxidative stress is a central feature of diabetic complications (Aihemaiti et al. 2024), this pathway may represent a potential mechanism through which MALAT1 contributes to diabetes-associated cellular dysfunction. In addition, MALAT1 has been linked to inflammatory responses involving the ASK1/p38/NLRP3 signalling axis under diabetic conditions (Lu et al. 2022; Puthanveetil et al. 2015; Zhang et al. 2017a, b).

Similarly, H19 has been implicated in cellular differentiation and metabolic signalling (Gabory et al. 2006). H19 also functions as a precursor of some microRNAs, including miR-675, and acts as a molecular modulator in several signalling pathways (Fan et al. 2019; Sanchez-Parra et al. 2018). Dysregulation of H19 has been associated with its function as a competing endogenous RNA (ceRNA), acting as a microRNA sponge and thereby influencing cellular proliferation, inflammation, epithelial-to-mesenchymal transition (EMT), oxidative stress and hepatic glucose production (Hernández-Aguilar et al. 2021; Wu and Huang 2023; Zhang et al. 2018). In addition, H19 has been shown to promote YAP1 dephosphorylation and activation, favouring TEAD-mediated transcription of downstream targets such as CTGF and CYR61 (Li et al. 2024). While relatively few studies have examined the relationship between H19 and YAP1 in the context of diabetes, both molecules have independently been associated with pathways involved in diabetic complications, including metabolic dysregulation (Wang et al. 2022; Zheng et al. 2017), fibrosis (Chen et al 2020; Wu and Huang 2023) and cellular stress responses (Guo et al 2021; Zhou et al. 2026). Consequently, a potential interaction between H19 and YAP1 may represent a biologically relevant mechanism deserving further investigation. Nevertheless, the structural basis of this association remains unclear.

Among intracellular signalling proteins associated with diabetes mellitus and its complications, Apoptosis Signal-regulating Kinase 1 (ASK1) and Yes-Associated Protein 1 (YAP1) have received considerable attention (Cheon and Cho 2019; Thakur et al. 2025). ASK1 is a stress-responsive mitogen-activated protein kinase kinase kinase (MAP3K5) involved in apoptosis, oxidative stress and inflammatory signalling through activation of the p38α/β and JNK1/3 pathways (Bunkoczi et al. 2007; Obsilova et al. 2021; Ogier et al. 2020). In contrast, YAP1 is a major downstream effector of Hippo signalling that regulates cell proliferation, survival and metabolic responses through interactions with TEAD transcription factors (Bae and Luo 2018; Shi et al. 2020; Wei et al. 2023a). Dysregulation of both proteins has been implicated in the pathogenesis of several disorders, including cancer, diabetes and cardiovascular diseases (Chen et al. 2022; Li and Huang 2023; Liu et al. 2025; Wei et al. 2023b).

Collectively, available evidence suggests that MALAT1 and H19 participate in signalling pathways associated with oxidative stress, inflammation and metabolic dysregulation. Moreover, MALAT1 has been linked to ASK1-related signalling, whereas H19 has been associated with YAP1 activation and Hippo pathway regulation, providing a biological rationale for investigating these RNA–protein pairs.

To further understand the role of lncRNA on protein regulation, this study aimed to in silico investigate the amino acid residues where the lncRNAs MALAT1 and H19 bind on ASK1 and YAP1 respectively, and their possible implications on their regulation within a diabetes framework. For this purpose, first, proteins and IncRNAs structures were modelled. Then, protein structural studies were performed to predict cavities, potential catalytic, allosteric sites and protein configuration was analysed with Equilibrium Molecular Dynamics studies (EMD). Finally, molecular docking studies were performed to predict the protein-lncRNA binding complexes and ASK1–MALAT1 and YAP1–H19 interfaces were analysed.

## Material and methods

### Molecule modelling and protein analyses

Protein sequences were obtained using UniProt, from the mitogen-activated protein kinase kinase (ASK1; Rattus norvegicus; 1381 Amino acids; EC: 2.7.11.25; UniProt ID: D3ZZH6) and the transcriptional coactivator (YAP1; *Rattus norvegicus*; 469 Amino acids; EC: 2.7.11.1; 469; UniProt ID: Q2EJA0). IncRNA sequences, namely MALAT (BC090353.1) and H19 (NR_027324.1), were obtained from GenBank. Both proteins and IncRNAs were modeled using AlphaFold 3 model in AlphaFold server (https://alphafoldserver.com/).

To assess the structural prediction of the models, the following confidence indicators were retrieved from AlphaFold confidence reports. The chain pair PAE min (PAE) is the lowest value in the predicted aligned error matrix between two specific chains (< 5 Å= very high confidence; 5–10 Å= moderate confidence; greater than 15 Å= very low confidence). The predicted template modelling score (pTM) measures the confidence in the global structure topology (a value > 0.5 indicates that the overall predicted fold and the arrangement of the protein is likely correct and structurally similar to the true biological structure), and the ranking score which is a weighted linear combination of ipTM and pTM [0.8 ipTM + 0.2 pTM]. Where: <0.5 indicates not or low confidence; 0.5–0.7 moderate confidence; 0.7–0.8 high confidence; 0.8–1.0 very high confidence).

### Protein analyses

To predict protein cavities and detect potential binding sites, Cavityplus was used (http://www.pkumdl. cn:8000/cavityplus/). Potential allosteric sites were predicted using AllositePro 2016 (https://mdl.shsmu.edu.cn/AST/). To analyze protein structural stability, Equilibrium Molecular Dynamics (EMD) studies were conducted in Gromacs (v2024.4; Abraham et al. 2025; Bekker et al. 1992). In brief, the protein model obtained from Alphafold was prepared using CHARMM-GUI (Jo et al. 2008). A water box 10 Å spaced from the protein edge was constructed. To represent the molecule force fields, the CHARMM36m force field was used (Huang et al. 2017), and the system was neutralized using NaCl ions (0•15 mM). A Langevin dynamics scheme was applied to maintain a temperature of 310 K (36.85ºC). In the minimization process, 2500 steps were used; for the NVT equilibration process, 5,000,000 steps (5 ns) were applied; and for the NPT production run, 50,000,000 steps (100 ns) were performed. The time step was set to 2 fs/step. The changes in the protein configuration during the EMD were analyzed in PyMOL (version 3.1.3.1; Schrödinger 2025) and compared to the initial structure (Alphafold original model). To assess the protein average structural deviation throughout the trajectory, the root–mean–square deviation (RMSD) was calculated with the RMSD plugin using VMD 1.9.4. To analyze the flexibility of each amino acid over time, the root–mean–squared–fluctuation (RMSF) was calculated using the Geo Meassures pluggin in PyMOL (https://pymolwiki.org/Geo_Measures_Plugin; Kagami et al. 2020). To evaluate protein conformational mobility, folding and stability, intramolecular hydrogen bonds (H–bonds) were analyzed using the Hydrogen Bonds analysis tool within VMD 1.9.4. The resulting simulation trajectories were plotted using Xmgrace software.

### Molecular docking studies

To predict the protein-lncRNA binding complexes between ASK1-MALAT1 and YAP1-H19, the HDOCK server was used (http://hdock.phys.hust.edu.cn/), using a hybrid algorithm combining template-free ab initio coupling and homology-based modelling. Global coupling was performed using a Fast Fourier Transform (FFT) search. The following metrics were retrieved from the top 10 models: i) docking score: evaluates the theoretical affinity and stability of the complex. The more negative the value means a more possible favorable binding model; ii) confidence score: indicates the binding likeliness of two molecules as Confidence score = 1.0/[1.0+e0.02*(Docking_Score+150)]. When the confidence score is above 0.7, it indicates that the two molecules are very likely to bind; and iii) ligand root mean square deviation (RMSD) indicates the conformational stability of the protein once docked. From the top 10 models, model 1 was selected due to having the most favorable docking score among all models, indicating the most stable binding free energy, and its interface residues (all of the residue pairs within 5.0 Å between the protein and the lncRNA) were retrieved.

## Results

### Structural validation of models

Both proteins ASK1 and YAP1 had PAE values <1 Å, indicating a high confidence in the amino acid relative positions and suggesting that nearby residues exhibited a defined spatial orientation (Table 1). However, the obtained chain pTM value (< 0.5) indicated a low confidence, suggesting that the structural prediction may have failed, which could be due to the presence of intrinsically disordered regions. The obtained ranking score values (0.5–0.7) showed a moderate confidence, suggesting that the overall folding is probably correct, but side chains could be inaccurate; this may also indicate the presence of loops and flexible regions.

**Table 1:** Protein and lncRNA structural confidence indicators

Modelling validation			
Model	Chain pair PAE min (Å)	Chain pTM	Ranking score
*Protein*			
ASK1	0.76	0.48	0.61
YAP1	0.76	0.18	0.62
*LncRNA*			
MALAT1	0.85	0.19	− 99.81
H19	0.84	0.20	0.20

Chain pair PAE min = (< 5 Å= very high confidence; 5–10 Å= moderate confidence; greater than 15 Å= very low confidence); Predicted template modelling score (pTM; > 0.5 indicates that the overall predicted fold and the arrangement of the protein is likely correct and structurally similar to the true biological structure); Ranking score (< 0.5 = not or low confidence; 0.5–0.7 = moderate confidence; 0.7–0.8 = high confidence; 0.8–1.0 =very high confidence)

Furthermore, the PAE values (< 1 Å) for both lncRNAs, MALAT1 and H19, showed a near-perfect atomic precision with high confidence in local folding (Table 1). However, the obtained chain pTM value (< 0.5) showed low confidence and suggests that although small regions are well modelled separately, their overall arrangement is a low-confidence estimate due to the way lncRNA folds on a large scale, or due to the large size of the molecule. The negative ranking score value (− 99.81) for MALAT1 indicated that the model had a massive physical collision of atoms or clashes. In contrast, for H19 the obtained positive ranking score value (< 0.5) showed that the model had very low overall confidence, because lncRNAs exhibit high intrinsic flexibility in solution. However, the positive value ruled out the possibility that the model experienced severe atomic clashes or a physical collapse.

### ASK1-MALAT1

#### ASK1 analyses

**Fig. 1:**
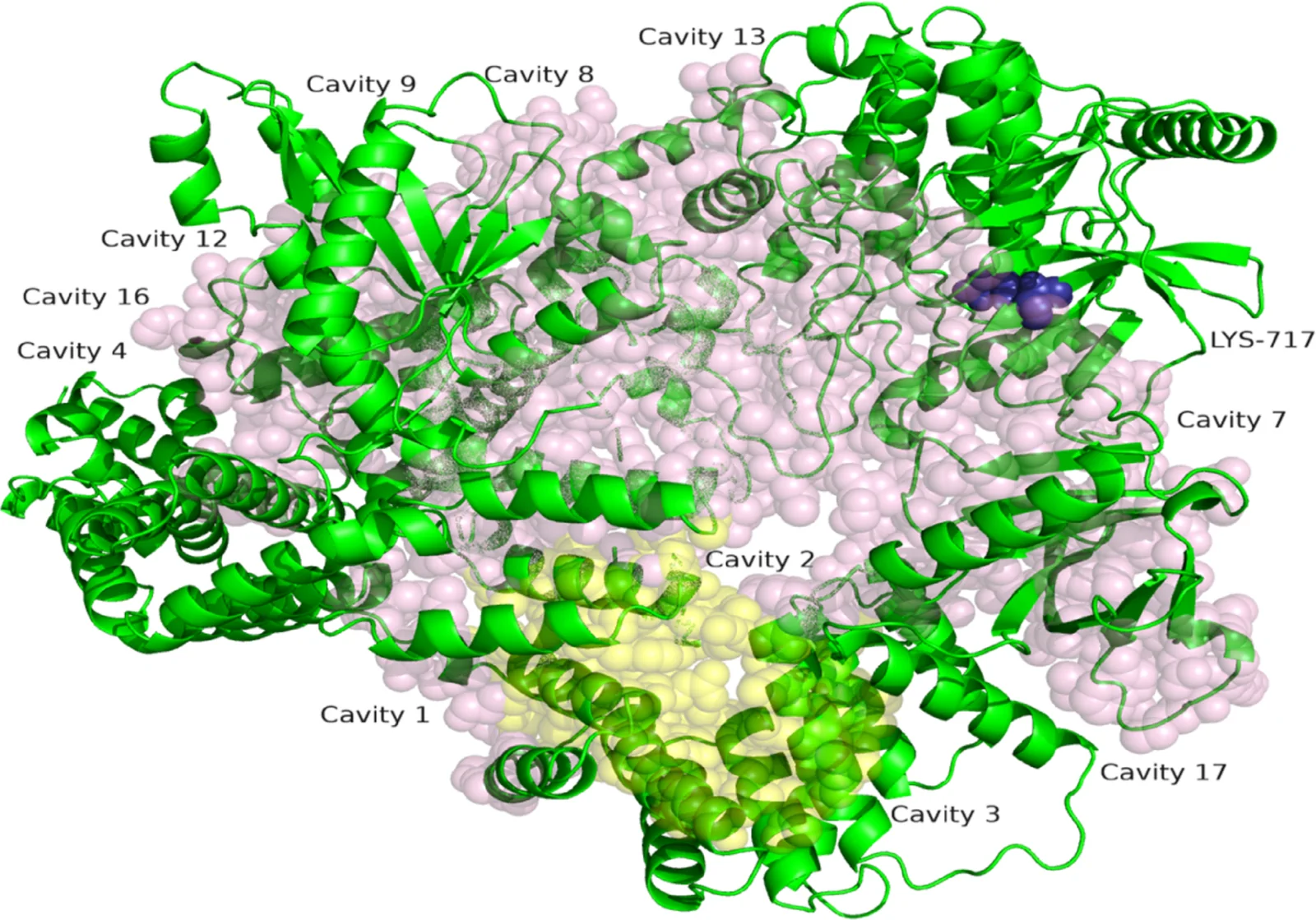
ASK1 cavities exhibiting high druggability. The main chain is depicted in green. Cavities with high drugability (1, 2, 13, 9, 4, 7, 12, 8, 16 and 17) are shown in light pink, whereas cavity 3 which exhibits the highest druggability score, is highlighted in yellow. The catalytic residue Lys717 is rendered in royal blue

**Fig. 2:**
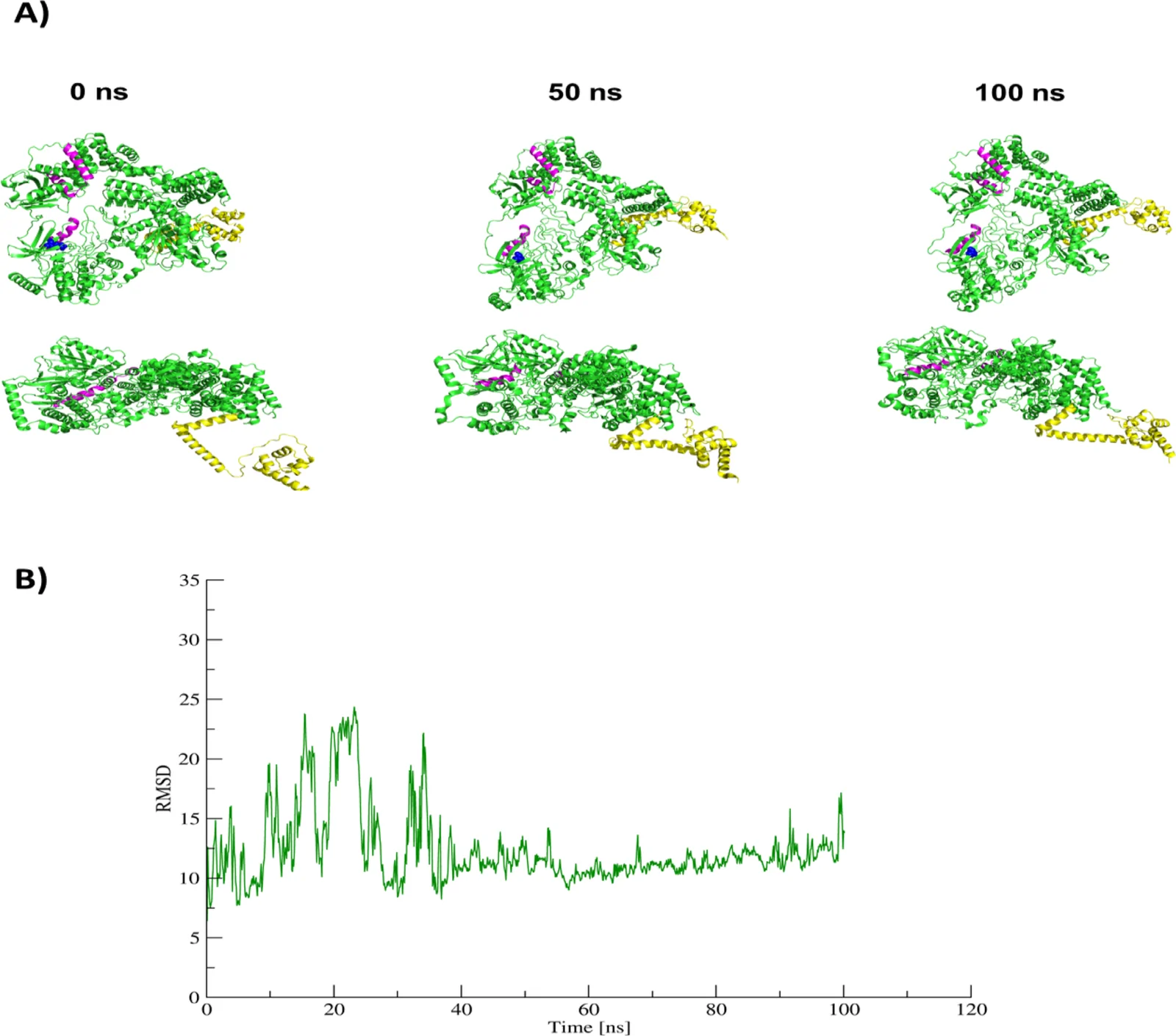
Equilibrium molecular dynamics trajectory of ASK1 over 100 ns. **A** Frontal and lateral views of ASK 1 (green) at different simulation intervals: the Initial state (0 ns); an intermediate transition (50 ns) wherein the ASK1 mobile region (yellow) folds towards the protein core, and the predicted allosteric site begins to undergo conformational separation (hot pink); and the final state (100 ns) showing the predicted allosteric site further separated and the mobile region folded. The catalytic residue Lys717 is depicted in royal blue; **B** Time-dependent root-mean-square deviation (RMSD) plot of the protein backbone during the 100 ns simulation trajectory

**Fig. 3:**
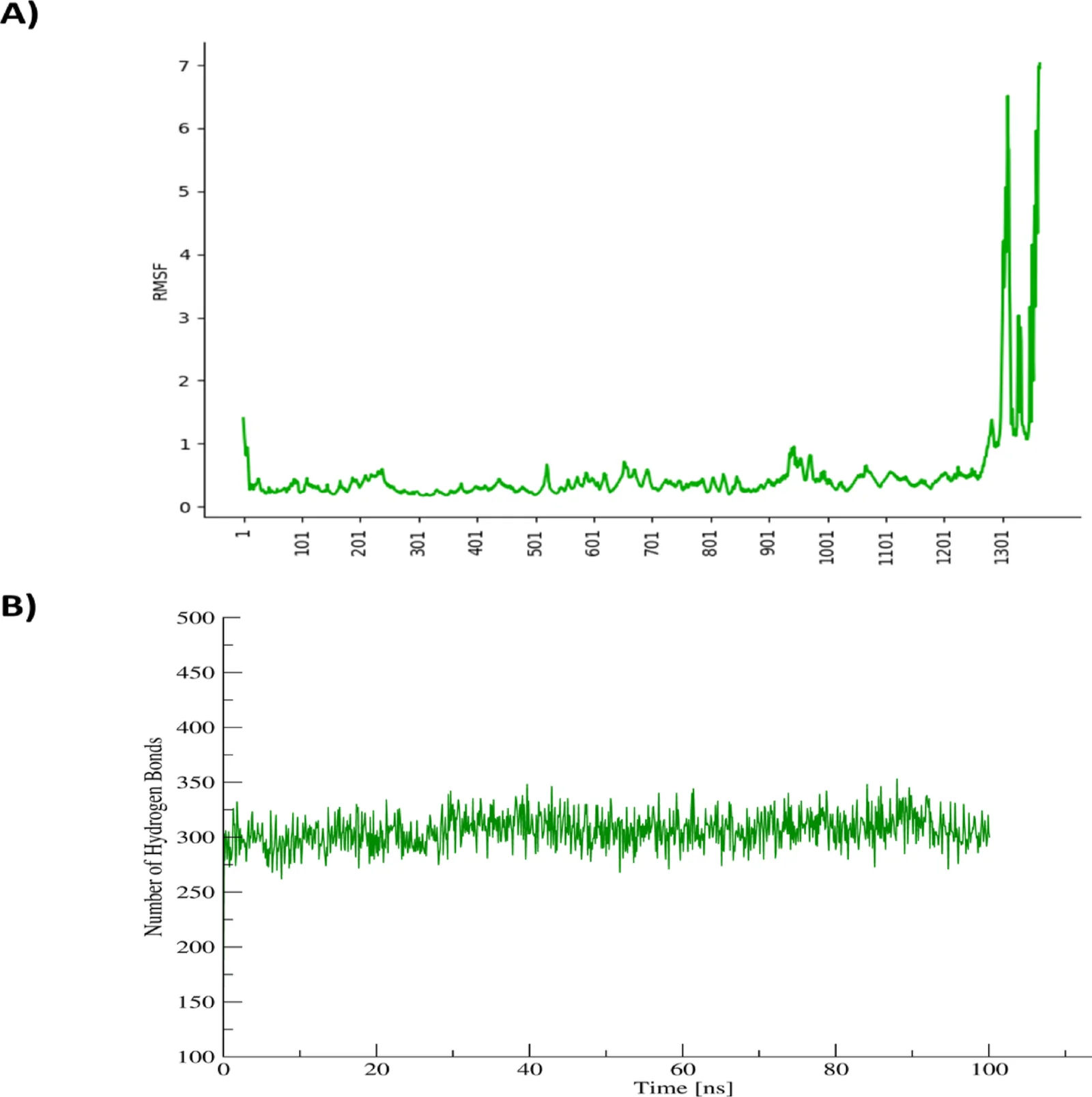
Equilibrium molecular dynamics trajectory of ASK1 over 100 ns. **A** Root-mean-square fluctuation (RMSF) profile of individual amino acid residues; **B** Time-dependent evolution of intramolecular hydrogen bonds

Sixty-one cavities were found along the ASK1 surface (Fig. 1). Eleven of these showed a strong druggability score (3, 1, 2, 13, 9, 4, 7, 12, 8, 16 and 17; Table 2). A pocket was identified as a potential allosteric site (AlloSite score 0•629) compromising cavities 1, 2, 3, 4, 7, 9 and 13. The catalytic residue Lys717 was located near the potential allosteric site in cavity 13 and showed the highest Pred Max pKd score, suggesting the best ligandability among all the potential binding sites. The EMD results showed that throughout the 100 ns simulation, the mobile region (residues Ser1248–Ala1381, yellow; Fig. 2A) in ASK1 folded towards the protein core. Currently, ASK1 underwent structural changes within the predicted allosteric site, which transitioned into a more open conformation (comprising residues His482–His514 and Ser707–His722), while the mobile region changed from a rigid, extended conformation to being folded towards the protein. Nevertheless, the final conformation revealed that the mobile region remained distant from the protein core. The RMSD showed that after an initial phase of high instability and substantial structural rearrangement lasting until 35 ns with peak values approaching 24 Å (Table 3; Fig. 2B), the system reached a steady state from 40 ns onwards, converging and stabilising between 10 and 12 Å. The RMSF revealed that residues 1300–1365, located within the C–terminal random coil region, exhibit an RMSF value greater than 2 Å, indicating that this is a highly flexible region (Fig. 3A).

**Table 2:** Cavities exhibiting high druggability in the ASK1 protein model

Cavity ID	Pred Max pKd	Pred Ave pKd	DrugScore
3	9.14	6.85	5171
1	08.01	6.78	5030
2	8.94	6.84	3735
13	11.85	6.68	2126
9	10.72	6.95	2004
4	9.51	6.87	1909
7	10.18	6.92	1797
12	11.66	6.87	1493
8	10.68	6.95	1357
16	11.20	6.46	1234
17	11.16	6.44	652

**Table 3:** (RMSD) and H–bonds values of equilibrium molecular dynamics (EMD) trajectories for the diabetes-related proteins ASK1 and YAP1

RMSD				
Protein	Mean	SD	Min	Max
ASK1	12.38	3.14	6.46	24.35
YAP1	35.24	4.80	1.32	39.57
		H–bonds		
ASK1	306.03	15.68	151	353
YAP1	60.17	10.97	33	94

The hydrogen bond analysis showed that the system maintained a constant profile with an average of 306.03 ± 15.68 interactions, confirming the conservation of structural integrity and stable intramolecular contacts within the protein (Fig. 3B).

#### ASK1–MALAT1 binding complexes

**Fig. 4:**
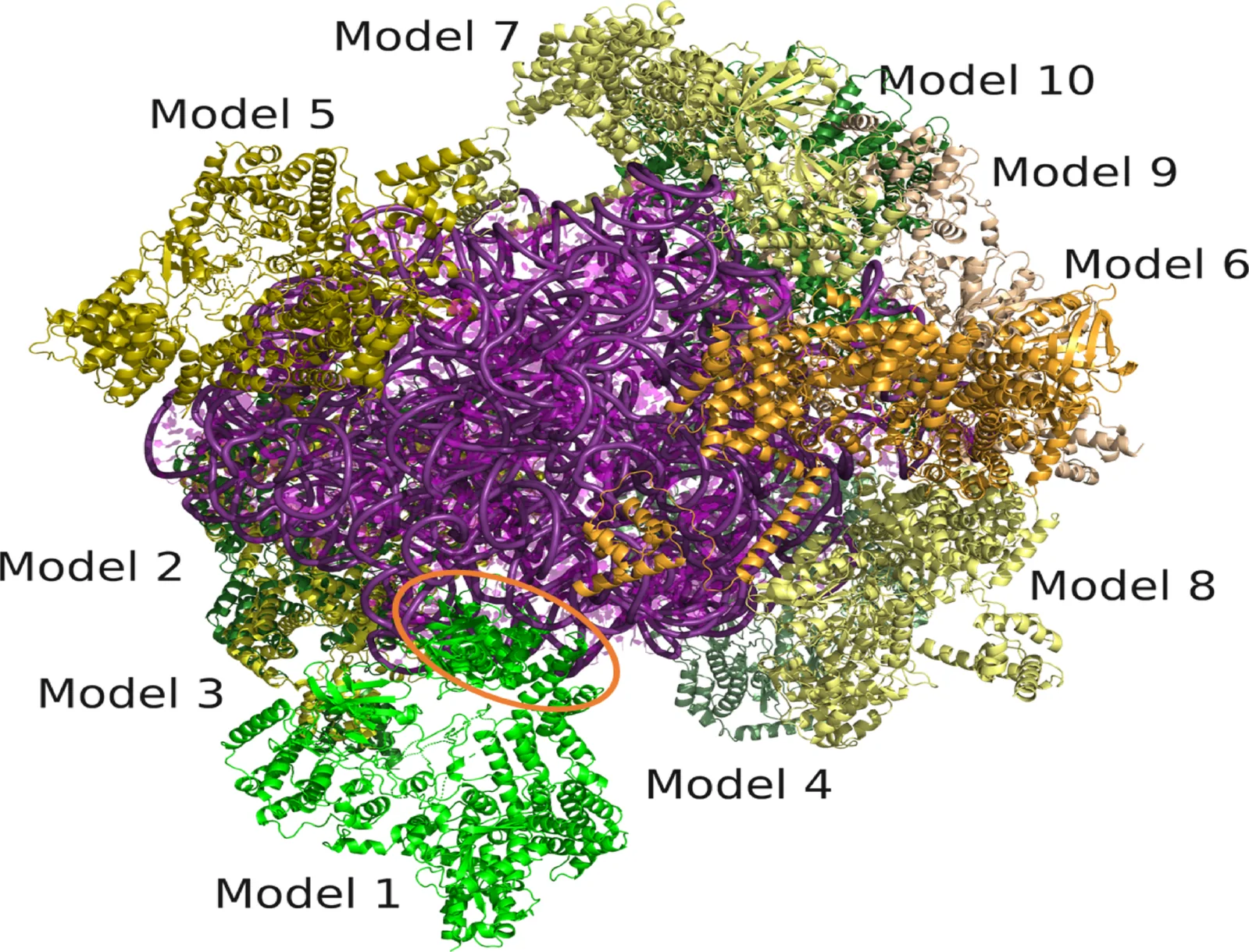
Predicted binding complexes of the ASK1-MALAT1 interaction obtained from molecular docking studies. ASK1 in Model 1 is rendered in green, whereas MALAT1 is depicted in purple. The orange circle indicates the binding interface of Model 1. Models 2 to 10 are shown in graduated shades of yellow and green

Molecular docking studies showed that Model 1 represented the best interaction pose for ASK1–MALAT1 complex registering the most favorable binding affinity with a docking score of − 404.46 and a confidence score of 0.9939 (Table 4; Fig. 4). The RMSD values for the ligand showed notable variability; Model 1 achieved an optimal balance high energetic stability and a structurally representative conformation (139.48 Å), whereas Model 5 presented the lowest structural deviation (76.52 Å).

**Table 4:** Molecular docking scores of the ASK1–MALAT1 complex

Model	Docking score	Confidence score	Ligand RMSD (Å)
1	− 404.46	0.9939	139.48
2	− 393.67	0.9924	171.26
3	− 381.26	0.9903	170.94
4	− 354.50	0.9835	196.68
5	− 350.23	0.9821	76.52
6	− 339.66	0.9780	129.26
7	− 330.14	9.9735	128.60
8	− 327.26	0.9719	179.22
9	− 325.22	0.9708	171.20
10	− 324.10	0.9702	156.29

#### ASK1–MALAT1 interface

**Fig. 5:**
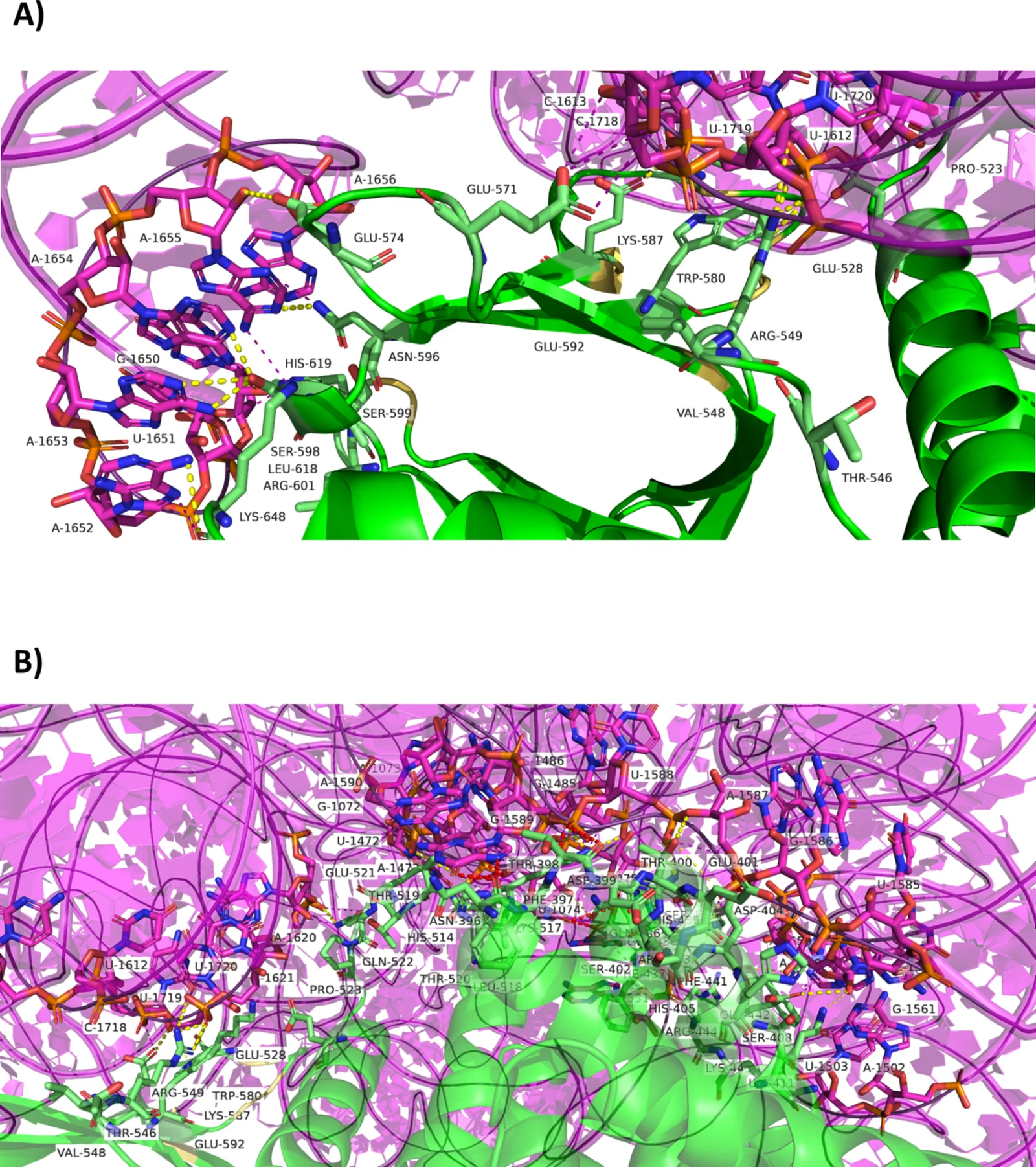
Amino acid residues and nucleotides involved at the Model 1 interface of the predicted binding complex for the ASK1-Malat1 interaction

Model 1, obtained from the molecular docking studies, showed that at the interface of the ASK1–MALAT1 complex (Fig. 5). ASK1 interacted via the following amino acid residues: 396–405, 408, 411, 434–439, 441–442, 444–445, 514, 517–523, 528, 546, 548–549, 571, 574, 580, 587, 592, 596, 598–599, 601, 618–619, 648 (Table 5). Whereas the MALAT1 lncRNA interacted via nucleotides 1072–1075, 1472–1473, 1485–1486, 1502–1503, 1530–1531, 1559–1562, 1585–1588, 1589–1590, 1612–1613,1620–1621, 1650–1656, 1718–1720.

**Table 5:** Interface amino acid residues and nucleotides within ASK1–MALAT1 complex

Protein	RNA	Distance (Å)
Asn396	A1590	4.013
Asn396	G1589	2.628
Phe397	G1589	3.386
Thr398	G1589	2.961
Asp399	G1589	4.975
Thr400	A1587	2.067
Thr400	G1586	4.489
Thr400	G1589	3.898
Thr400	U1588	2.419
Glu401	A1587	2.633
Glu401	G1586	3.64
Ser402	A1587	4.522
Arg403	A1587	4.971
Arg403	G1589	4.993
Arg403	U1588	3.045
Asp404	A1587	1.446
Asp404	G1586	3.404
His405	G1586	3.573
His405	U1585	4.379
Ser408	G1586	4.963
Lys411	C1562	4.872
Lys411	G1561	3.707
Gly434	G1485	4.587
His435	G1561	4.914
Gln436	U1074	3.987
Phe437	A1531	4.692
Glu438	A1530	2.661
Glu438	A1531	4.569
Glu438	U1074	3.019
Glu438	U1075	3.281
Ser439	A1560	3.94
Phe441	A1502	4.133
Phe441	A1559	4.796
Phe441	A1560	2.747
Phe441	U1503	3.211
Glu442	A1560	4.138
Glu442	G1561	3.152
Arg444	A1531	4.663
Lys445	A1502	4.062
His514	G1589	3.638
Lys517	A1073	4.084
Lys517	C1486	3.293
Lys517	G1485	3.152
Lys517	U1074	2.101
Leu518	U1074	2.811
Thr519	A1073	2.245
Thr519	G1072	3.977
Thr519	U1074	2.488
Thr520	A1073	4.969
Glu521	A1073	3.826
Glu521	A1473	4.02
Glu521	A1620	4.722
Glu521	U1472	3.037
Gln522	A1620	3.431
Gln522	C1621	3.092
Pro523	A1620	4.243
Glu528	U1719	4.335
Thr546	U1612	4.882
Val548	C1613	4.586
Arg549	U1719	3.597
Arg549	U1720	2.449
Glu571	C1613	3.025
Glu574	A1655	3.309
Trp580	C1718	3.81
Trp580	U1719	2.791
Trp580	U1720	4.429
Lys587	C1718	4.357
Lys587	U1719	4.014
Glu592	C1718	2.844
Asn596	A1654	4.338
Asn596	A1655	2.409
Ser598	A1652	4.742
Ser598	A1653	2.449
Ser598	A1654	2.587
Ser598	A1655	4.698
Ser598	U1651	4.765
Ser599	A1655	4.812
Arg601	U1651	4.457
Leu618	U1651	4.558
His619	A1654	4.526
His619	A1655	3.614
His619	A1656	4.371
His619	G1650	2.467
His619	U1651	3.188
Lys648	A1652	3.144
Lys648	A1653	4.61

### YAP1–H19

#### YAP1 analyses

**Fig. 6:**
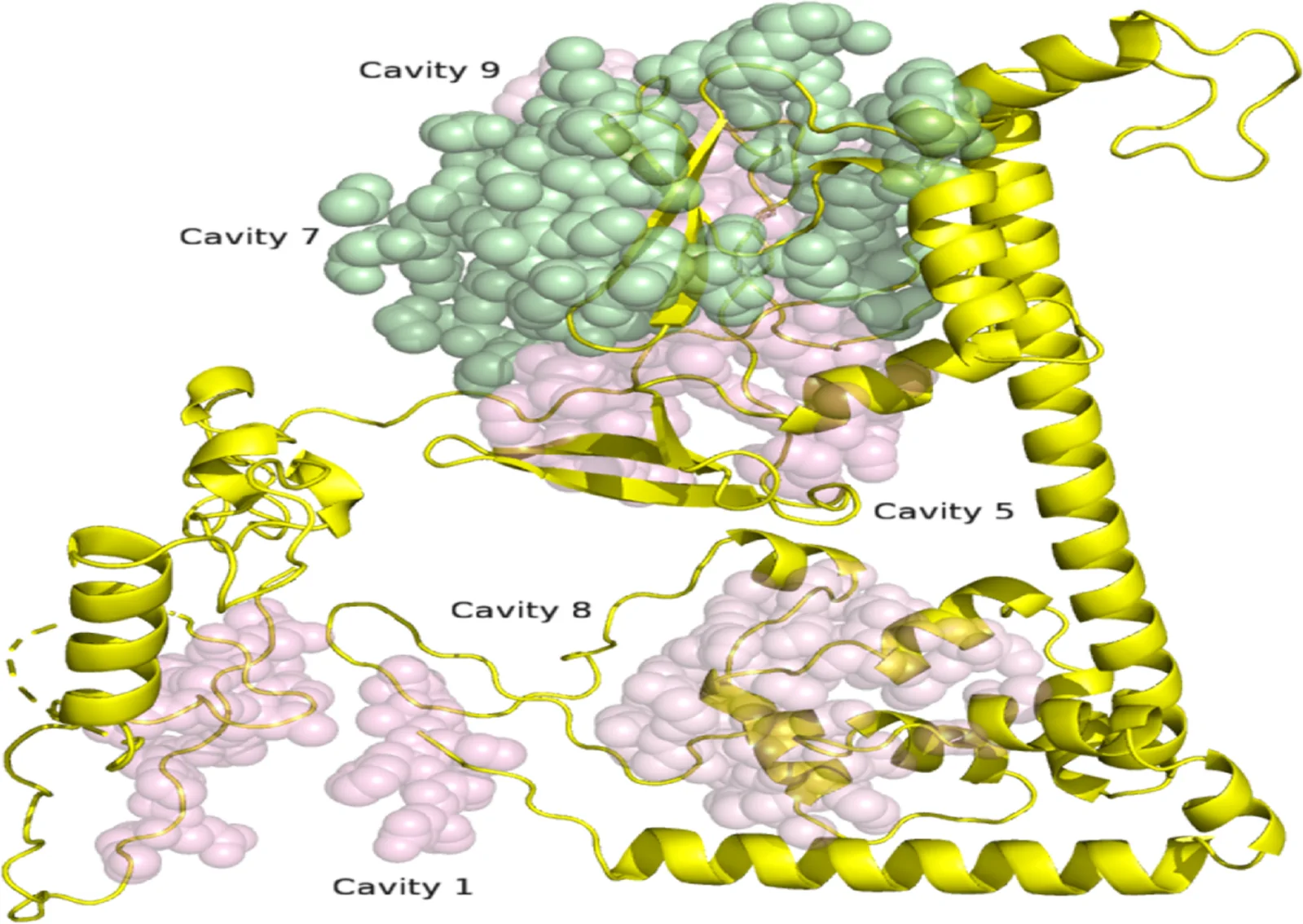
YAP1 cavities exhibiting high druggability. The main chain of YAP1 is depicted in yellow. Cavities with high druggability are shown in light pink, whereas cavity 7, which exhibits the highest druggability score, is highlighted in green

**Fig. 7:**
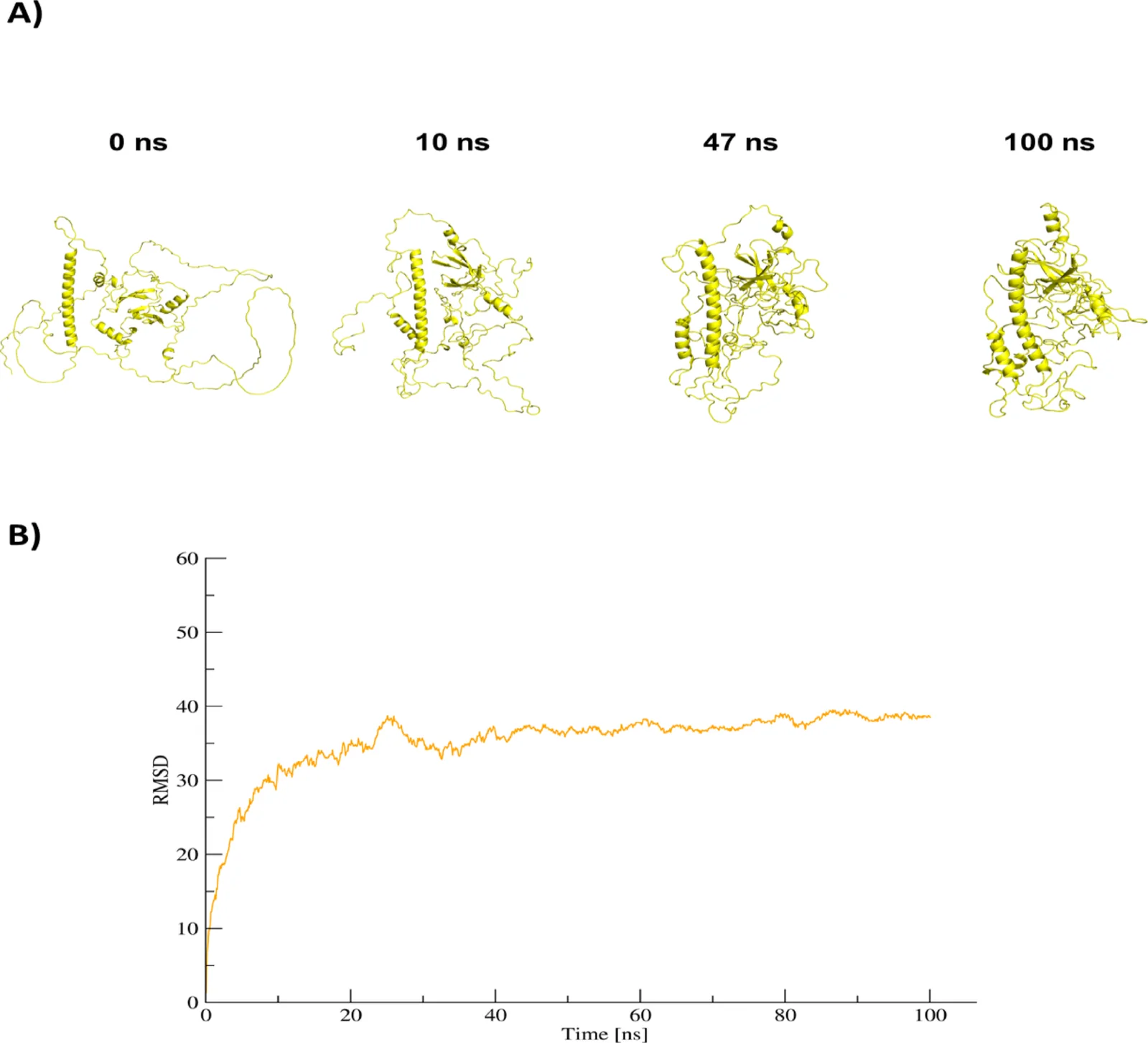
Molecular dynamics trajectory of YAP1. **A** Conformational transitions of YAP1 during 100 ns simulation. Initial state (0 ns) representing the relaxed conformation where the main alpha helix is maintained alongside regions of random coil. At the intermediate transition state (10 ns), the random loops approach both each other and the main alpha helix. By the intermediate transition state at 47 ns, the random loops have compacted towards the protein core. The final conformation (100 ns) showed that the protein structural arrangement remains stable until the end of the EMD trayectory. **B** Time-dependent root-mean-square deviation (RMSD) plot of the protein backbone during the 100 ns simulation trajectory, showing conformational changes of the protein with respect to the initial state

**Fig. 8:**
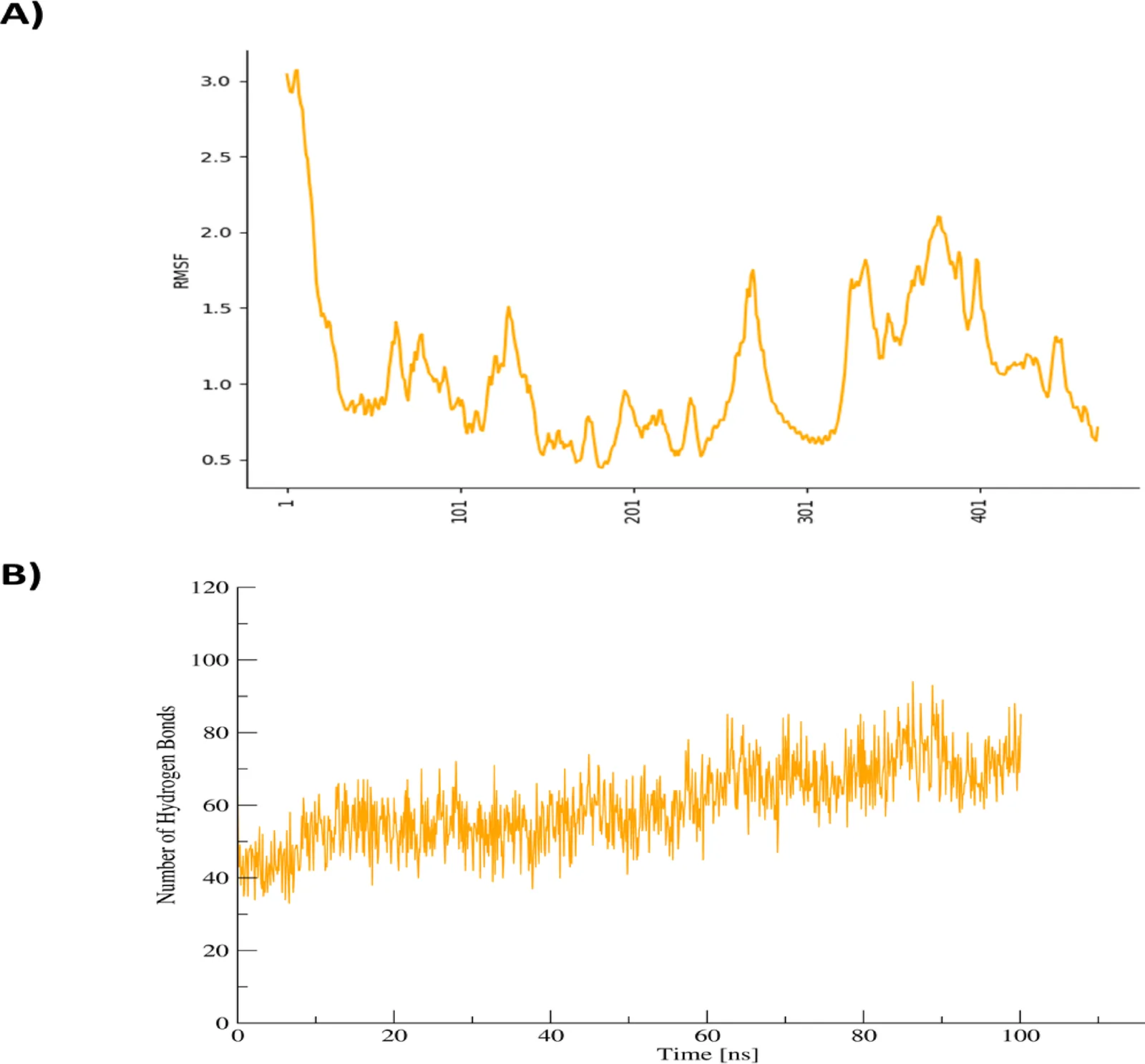
Equilibrium molecular dynamics trajectory of YAP1 over 100 ns. **A** Root-mean-square fluctuation (RMSF) profile of individual amino acid residues; **B** Time-dependent evolution of intramolecular hydrogen bonds

The surface of YAP1 showed 18 cavities, six of which showed a strong druggability score (Table 6; Fig. 6). No potential allosteric sites were identified. Cavity 7 exhibited the highest druggability score and Pred Max pKd. The EMD results showed that throughout the 100 ns simulation (Table 3), YAP1 underwent compaction, aligning two alpha helices (residues Gly279–Leu318 with Gly44–Asn59). A pair of antiparallel beta sheets attracted each other and arranged themselves behind the two alpha helices. The final conformation exhibited no structural changes, whereas the random loops formed new alpha helices while the other sections clustered at the protein core (Fig. 7A). The RMSD analysis showed that after an initial equilibration phase during the first 25 ns, the system stabilises from 40 ns onwards, maintaining a minimal and sustained fluctuations around 38 Å until the end of the 100 ns trajectory (Fig. 7B). The RMSF analysis showed that YAP1 exhibited stable regions within its core (residues 140–190 and 280–310), which remained rigid with fluctuations of less than 0.7 Å. Conversely, the *N*-terminus displayed high initial mobility (~ 3 Å), accompanied by multiple peaks of moderate and intermittent flexibility distributed throughout the remainder of the sequence (Fig. 8A).

**Table 6:** Cavities exhibiting high druggability in the YAP1 protein model

Cavity ID	Pred Max pKd	Pred Ave pKd	DrugScore
7	11.78	6.66	2792
4	11.06	6.97	2029
1	8.05	6.78	1601
5	11.08	6.98	1534
8	11.45	6.54	872
9	11.43	6.54	663

The hydrogen bond analysis showed that the system exhibited a progressive increase and marked variations over time, registering an average of 60.17 ± 10.97 hydrogen bonds. This suggests a constant and dynamic rearrangement of the intramolecular interactions within YAP1 (Fig. 8B).

#### YAP1–H19 binding complexes

**Fig. 9:**
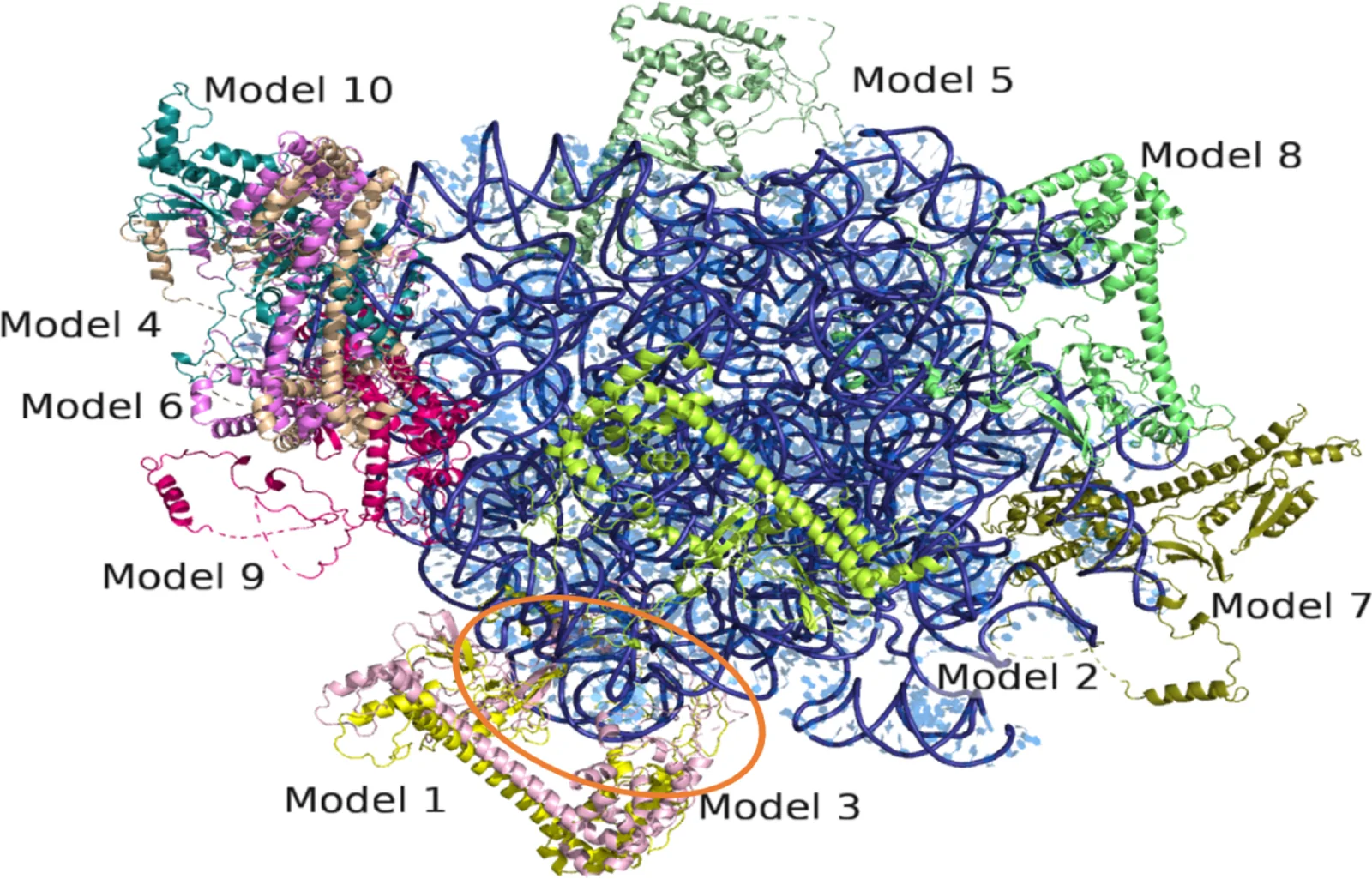
Predicted binding complexes of the YAP1-H19 interaction obtained from molecular docking studies. YAP1 in Model 1 is rendered in yellow, whereas H19 is depicted in dark blue. The orange circle indicates the binding interface of Model 1. Models 2 to 10 are shown in other colors

Molecular docking studies of the YAP1–H19 complex (Fig. 9) showed that Model 1 exhibited the lowest docking score and the highest confidence value (− 395.41 and 0.9927, respectively; Table 7) closely followed by Models 2 and 3, which also showed a high probability of interaction (confidence scores >0.99). Regarding the ligand RMSD, Model 3 showed the lowest value (1.28 Å), even when compared to that obtained for Model 1 (15.78 Å) suggesting a highly conserved native conformation.

**Table 7:** Molecular docking scores of the YAP1–H19 complex

Model	Docking score	Confidence score	Ligand RMSD (Å)
1	− 395.41	0.9927	15.78
2	− 384.83	0.9910	96.94
3	− 380.84	0.9902	1.28
4	− 330.10	0.9735	128.84
5	− 319.57	0.9674	169.66
6	− 315.12	0.9645	131.69
7	− 310.35	0.9611	159.53
8	− 303.80	0.9559	164.94
9	− 297.93	0.9507	105.42
10	− 297.54	0.9503	145.19

#### YAP1–H19 interface

**Fig. 10:**
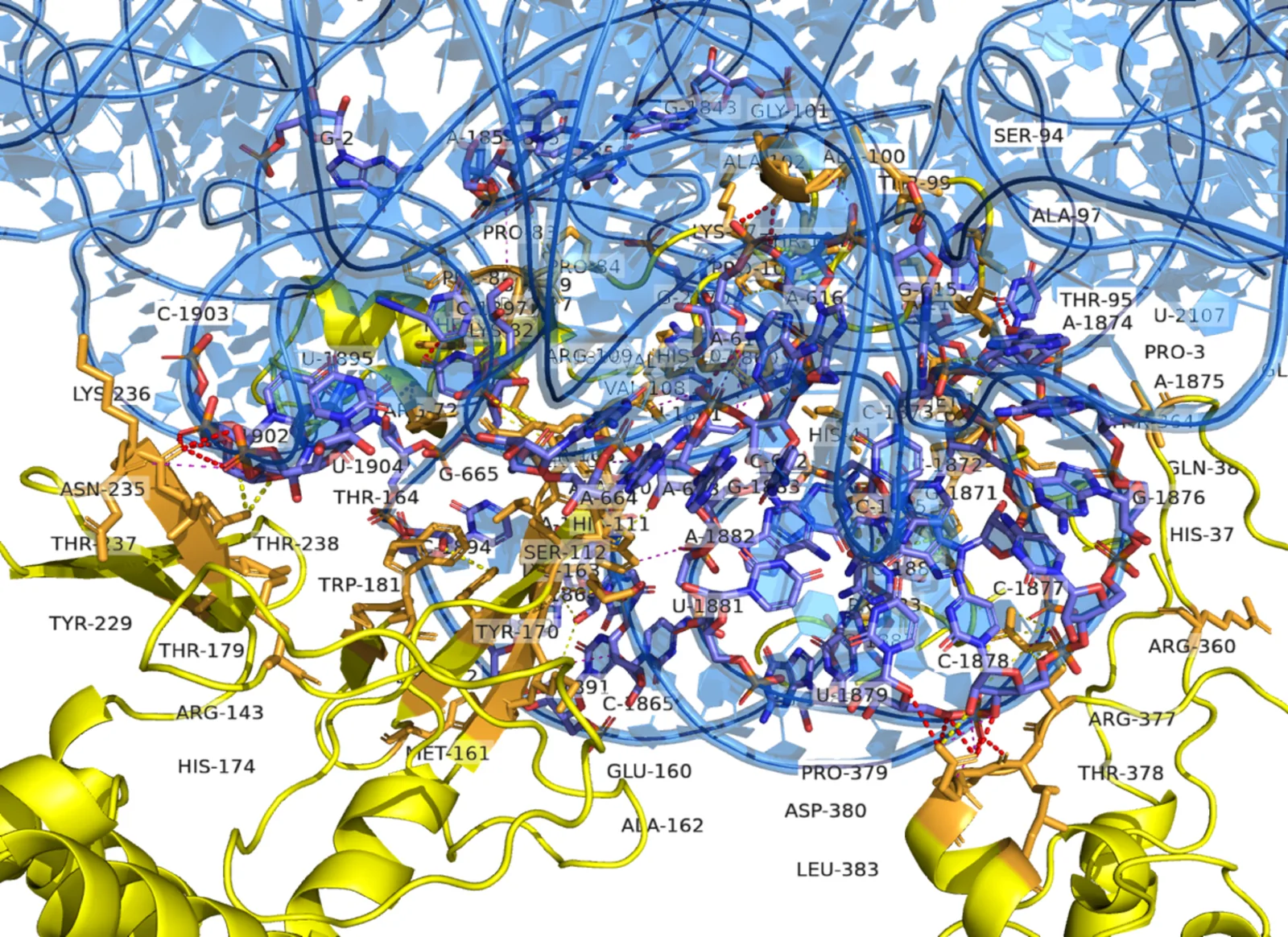
Amino acid residues and nucleotides involved at the Model 1 interface of the predicted binding complex for the YAP1-H19 interaction

Model 1, obtained from the molecular docking studies, showed that at the interface of the YAP1–H19 complex (Fig. 10), YAP1 interacted via the following amino acid residues: 1–3, 5, 33, 37–39, 41–42, 72, 77, 79–84, 87, 94–95, 97, 99–102, 104–105, 107–112, 143, 160–166, 170, 172, 174, 179, 181, 229, 235–238, 360, 364, 377–380, 383; whereas the H19 lncRNA interacted with the following nucleotides: 2, 615–617, 662–665, 1843, 1854–1856, 1860–1861, 1863–1865, 1871–1876, 1877–1879, 1881–1883, 1885–1888, 1891–1892, 1894–1897, 1902–1904 and 2107–2108 (Table 8).

**Table 8:** Interface residues and nucleotides within YAP1–H19 complex

Protein	RNA	Distance (Å)
Met1	C1872	4.493
Met1	C1885	4.693
Met1	G1871	4.772
Glu2	A2108	4.592
Glu2	U2107	3.059
Pro3	C1886	4.625
Gln5	A2108	2.76
Gln5	U2107	3.097
Pro33	G1887	4.997
Pro33	G1888	3.474
His37	C1886	4.178
His37	G1887	3.245
Gln38	C1885	4.545
Gln38	C1886	2.886
Gln38	G1887	3.29
Val39	C1886	4.159
Val39	G1887	2.828
His41	A1860	3.3
His41	U1861	3.458
Val42	A2108	3.117
Arg72	U1895	4.809
Pro77	C1856	4.13
Ser79	C1855	3.728
Ser79	C1856	3.928
Phe80	C1855	3.689
Phe80	G2	4.301
Phe81	U1896	3.402
Lys82	U1896	3.562
Pro83	A1854	4.33
Pro83	C1897	3.483
Pro83	U1896	2.334
Pro84	A1854	3.523
Lys87	G1843	4.259
Ser94	C1873	4.958
Thr95	A1874	2.989
Thr95	C1873	3.621
Ala97	A1874	3.702
Ala97	G615	4.206
Thr99	A616	4.322
Thr99	G615	4.471
Ala100	A616	3.472
Gly101	A616	2.801
Gly101	A617	3.172
Ala102	A616	4.89
Ala102	A617	4.706
Thr104	A616	2.362
Thr104	A617	4.582
Pro105	A616	3.892
His107	A616	3.249
His107	A617	4.028
His107	A663	1.907
His107	C662	2.626
Val108	A663	3.716
Val108	A664	4.616
Arg109	A663	4.122
Arg109	A664	3.142
Arg109	G665	3.87
Ala110	A663	2.16
Ala110	A664	2.317
His111	A1882	2.795
His111	A663	3.923
His111	A664	4.664
His111	U1881	4.5
Ser112	A664	4.411
Arg143	C1903	4.4
Glu160	G1892	4.656
Glu160	U1864	4.741
Glu160	U1891	3.521
Met161	C1865	3.375
Met161	U1864	3.561
Ala162	C1865	4.327
Ala162	U1864	3.221
Lys163	A1863	4.612
Lys163	A1882	4.458
Lys163	C1865	1.807
Lys163	G1883	4.201
Lys163	U1864	2.757
Thr164	U1864	3.644
Thr164	U1896	4.106
Ser165	U1864	4.618
Ser165	U1896	3.891
Ser166	C1897	3.487
Ser166	U1896	3.304
Tyr170	C1894	3.354
Leu172	G1892	4.965
His174	G1892	3.858
His174	U1891	4.41
Thr179	C1894	4.607
Trp181	U1896	4.826
Tyr229	U1902	4.755
Asn235	U1904	3.917
Lys236	C1903	2.858
Lys236	U1902	4.977
Lys236	U1904	3.113
Thr237	C1903	3.317
Thr237	U1904	2.316
Thr238	C1903	2.842
Thr238	U1902	2.628
Arg360	G1871	4.856
Thr364	A1875	4.021
Thr364	G1876	4.912
Arg377	C1877	4.997
Arg377	C1878	2.741
Arg377	U1879	3.115
Thr378	C1878	4.344
Thr378	U1879	2.331
Pro379	U1879	3.27
Asp380	C1878	2.618
Asp380	U1879	2.618
Leu383	U1879	4.579

## Discussion

Long non-coding RNAs (lncRNAs) are recognised as important regulators of multiple cellular processes through their interactions with proteins, and dysregulation of these interactions has been implicated in several pathological conditions (Li et al. 2025; Licatalosi and Darnell 2010; Márton et al. 2025; Moran et al. 2012). Previous studies have shown that lncRNAs may influence protein function through direct molecular interactions, including binding to regulatory regions involved in enzymatic activity and signalling processes (Li 2023; Wang et al. 2025).

In the present study, computational analyses predicted potential interactions between MALAT1 and ASK1, as well as between H19 and several functional regions within YAP1. These predicted interaction patterns were located near regions associated with allosteric regulation and protein–protein interaction interfaces involved in diabetes-related signalling pathways. Accordingly, the following sections discuss the potential biological relevance of these predicted lncRNA–protein associations in the context of previously reported experimental evidence.

### ASK1-MALAT1 complex

*Predicted MALAT1–ASK1 interaction and biological relevance*: Predicted MALAT1–ASK1 interaction and biological relevance. Computational analyses predicted a potential interaction between MALAT1 and ASK1 involving residues located within the *N*-terminal regulatory region (between 396–648) of ASK1 rather than within its catalytic kinase domain. These findings suggest that MALAT1 may associate with regulatory surfaces of ASK1; however, the present data do not demonstrate any effect on ASK1 activation or downstream signalling pathways.

Although no previous studies have investigated a direct physical interaction between MALAT1 (Ashjari et al. 2022; Hu et al. 2017; Taylor et al. 2024) and ASK1 (Demir et al. 2021; Roy 2025; Wang et al. 2015a, b), both molecules have been independently implicated in biological processes relevant to diabetic complications, including oxidative stress, inflammation, endoplasmic reticulum stress, and apoptosis. ASK1 functions as a central stress-responsive kinase that activates the JNK and p38 MAPK pathways in response to cellular stress (Hayakawa et al. 2012), whereas MALAT1 has been reported to modulate inflammatory and apoptotic responses in multiple experimental models (Li et al. 2022; Lin et al. 2019). Furthermore, MALAT1 has been associated with activation of the ASK1/p38 signalling axis during diabetic retinopathy (Lu et al. 2022), suggesting that both molecules may participate in related signalling networks.

To the best of our knowledge, no previous studies have examined the possibility of a direct MALAT1–ASK1 interaction. Therefore, the interface predicted here may represent a previously unrecognized point of contact between two molecules that converge in stress-response pathways associated with diabetes. Nevertheless, the present computational analyses do not establish a mechanistic relationship between MALAT1 binding and ASK1 activity, and any functional implications remain speculative pending experimental validation.

*From a pharmacological perspective*: The predicted interface could serve as a starting point for future studies aimed at evaluating whether modulation of MALAT1–ASK1 association influences stress-responsive signalling pathways. However, the present study was not designed to assess druggability or therapeutic potential, and these possibilities require further investigation.

### YAP1-H19 complex

*Predicted H19–YAP1 interactions near conserved regulatory regions*: Computational analyses predicted that H19 may be capable of interacting near several regions within YAP1, including the TEAD-binding domain (TBD) and intrinsically disordered regions known to contain regulatory phosphorylation sites (Newcombe et al. 2022) in other species (UniProt Consortium 1995a, 1995b, 2019). These regions are involved in the control of YAP1 localisation, stability and transcriptional activity within the Hippo signalling pathway (Fu et al. 2022). It must be emphasised, however, that the present computational data indicate only potential spatial proximity; they do not demonstrate that H19 binding affects any of these processes.

Because functional characterisation of YAP1 residues in rat models remains limited, orthologous sequence alignment was performed using experimentally characterised human (Persson and Sonnhammer 2023) and mouse (Persson and Sonnhammer 2023) YAP1 proteins. This analysis revealed that several predicted H19 interaction sites are located near conserved regulatory residues. Notably, rat Ser112 corresponds to human Ser127, one of the best-characterised phosphorylation sites within YAP (Zindel et al. 2019), whereas rat Thr104 corresponds to human Thr119, a residue implicated in additional regulatory phosphorylation events (Zhang et al. 2025). The proximity of predicted contacts to these sites raises a structural hypothesis worth testing, namely whether H19 binding could influence access to these residues. No functional conclusion is drawn from proximity alone.

The TEAD-binding domain is essential for YAP1-mediated transcriptional activity, enabling interactions with TEAD family transcription factors and promoting the expression of genes involved in cell proliferation, survival, differentiation and tissue growth (Li et al. 2010; Pocaterra et al. 2020; Tian et al. 2010). Structural studies have shown that stable YAP1–TEAD complexes are formed through *N*-terminal regions encompassing residues 50–114, 50–159 and 50–171, with residues located within the 86–100 region contributing substantially to the interaction interface (Li et al. 2010). In the present study, several predicted H19 contacts were identified within regions adjacent to these *N*-terminal YAP1 segments. These predictions do not demonstrate interference with TEAD binding. They indicate, at most, that the predicted H19-binding surface on YAP1 is spatially close to, but not necessarily overlapping with, the TEAD-binding interface.

Attention was paid to the proximity of predicted H19 contacts to rat Ser112, corresponding to human Ser127. Experimental studies have demonstrated that Ser127 is a major phosphorylation site targeted by LATS1/2 kinases and plays a central role in Hippo pathway regulation (Hao et al. 2008; Zhao et al. 2007). Phosphorylation at this residue promotes binding to 14-3-3 proteins, resulting in cytoplasmic retention and functional inhibition of YAP1. Mutation of Ser127 disrupts 14-3-3 binding, reduces cytoplasmic sequestration and enhances YAP1 nuclear localisation and transcriptional activity (Zhao et al. 2007). Because several predicted H19 contacts are located near the orthologous rat residue, one can formulate the following structural hypothesis: H19 may bind in a manner that could, in principle, influence the accessibility of Ser112. However, the present computational analyses provide no evidence that H19 directly affects phosphorylation events, 14-3-3 recruitment or YAP1 localisation. It should also be noted that most studies characterising Ser127 function have been conducted in non-diabetic experimental systems, particularly in cancer and developmental biology models. Therefore, the relevance of these mechanisms in diabetes remains to be established experimentally.

Additional contacts were identified near rat Thr104, corresponding to human Thr119. Although this residue has received less attention than the well-characterised Ser127 regulatory site, previous studies have shown that Thr119 can be phosphorylated by kinases such as CDK1, CDK8 and AMPK under specific cellular conditions (Kang et al. 2023; Wang et al. 2015a, b; Yang et al. 2013; Zhou et al. 2018). These observations suggest that Thr119 may participate in the regulation of YAP1 activity, linking this residue to processes including cell-cycle progression (Zhu and Zhao, 2014), stress responses and metabolic signalling. The proximity of predicted H19 contacts to this conserved regulatory site further supports the structural relevance of the predicted interaction. However, the present computational analyses do not provide evidence that such interactions influence Thr119 phosphorylation or any downstream biological process. Further experimental studies will be required to determine the functional significance of this predicted association.

Previous experimental studies have also reported functional relationships between H19 and YAP1. For example, H19 has been shown to promote YAP1 dephosphorylation and activation, favouring TEAD-mediated transcription of downstream targets such as CTGF and CYR61 (Tao et al. 2021). Although this evidence was not generated in diabetic models, it supports the notion that H19 and YAP1 may participate in shared regulatory networks. The interaction interfaces predicted in the present study could represent structural regions through which such experimentally observed functional relationships occur, but this remains a hypothesis that requires direct testing.

*O-GlcNAcylation as additional metabolic context*: YAP1 activity is also regulated by O-linked β-*N*-acetylglucosamine (O-GlcNAc) modification, a nutrient-sensitive post-translational mechanism frequently altered under diabetic conditions (Slawson et al. 2010). Previous studies have shown that O-GlcNAcylation of YAP1 at Ser109 can influence its localisation and transcriptional activity (Peng et al. 2017). Although the predicted H19–YAP1 interfaces identified in this study do not directly overlap with this residue, these findings provide additional metabolic context for interpreting YAP1 regulation in diabetes. The present computational analyses were not designed to evaluate interactions between H19 binding and O-GlcNAcylation; therefore, any potential relationship between these regulatory mechanisms remains to be experimentally investigated.

*Mechanobiological context*: YAP1 functions as a key mechanotransducer that integrates signals derived from extracellular matrix stiffness and cytoskeletal dynamics into transcriptional responses (Shen et al. 2025). Altered YAP1 activity has been implicated in several diabetes-associated processes, including fibrosis (Zhang et al. 2020), inflammation (Ortillon et al. 2021) and cell survival (Yuan et al. 2016; Zhao et al. 2025.). The proximity of the predicted H19 interaction sites to regulatory regions of YAP1 supports the biological relevance of further investigating this interaction in diabetic contexts. However, the present computational data do not provide evidence that H19 directly influences mechanotransduction or YAP1-dependent cellular responses.

*Pharmacological relevance*: Although the present study is exploratory and entirely computational, the predicted RNA–protein interfaces may have potential value for future target-validation efforts. If experimentally confirmed, the identified interaction regions could help define structural sites involved in MALAT1–ASK1 and H19–YAP1 association. Such information may be useful for the future development of strategies aimed at modulating RNA–protein interactions, including antisense oligonucleotides, RNA-targeting approaches or small molecules designed to disrupt or stabilise specific molecular interfaces. In the context of diabetes, these predicted interactions provide testable hypotheses that may contribute to understanding how lncRNAs participate in signalling pathways associated with oxidative stress, cellular survival and Hippo pathway regulation.

## Limitations of the study

Furthermore, the in silico analysis limitations in this study arose from the high conformational flexibility and rotational entropy of lncRNAs, which introduced significant uncertainty in regions of macrostructural disorder, mainly evidenced by low overall confidence metrics with pTM values below 0•5, as well as the occurrence of severe atomic clashes reflected in the negative ranking score for MALAT1. Moreover, in this study MDE studies were restricted to unliganded proteins, omitting the coupled protein-lncRNA complexes. The accumulation of sampling bias in the conformational space of the interface nucleotides precludes the thermodynamic convergence of the entire molecular system within standard atomistic simulation timescales. This is due to the sheer size of the molecular complexes, their high thermodynamic instability, and the lack of rigorous parametrization in classical force fields (such as CHARMM36m) to simulate large-scale, coupled conformational transitions of polyanionic macromolecular RNAs.

## Conclusion

Overall, the present findings suggest that H19 may associate with regions of YAP1 located near conserved regulatory residues and functionally important interaction surfaces. Nevertheless, these observations are derived exclusively from computational modelling and should be interpreted as structural hypotheses, not as evidence of direct functional regulation. They warrant further experimental investigation using approaches such as RNA immunoprecipitation, CLIP-based assays and RNA pull-down experiments to determine whether these predicted interactions occur under physiological conditions and, if so, whether they influence YAP1 or ASK1 signalling. Importantly, although many of the regulatory residues discussed here have been extensively characterised in human and murine systems, most available evidence originates from non-diabetic biological contexts. Consequently, the potential relevance of these predicted interaction interfaces to diabetes-associated signalling remains inferential and requires direct experimental validation in diabetic models. This study identified predicted amino acid residues involved at the modelled interfaces of the ASK1–MALAT1 and YAP1–H19 complexes in *Rattus norvegicus*. Further in vitro and in vivo validation is required to elucidate their full biological relevance.

## Data Availability

No datasets were generated or analysed during the current study.
